# Beneficial effects of ultrafine bubble shower on a mouse model of atopic dermatitis

**DOI:** 10.3389/fimmu.2024.1483000

**Published:** 2024-12-26

**Authors:** Ayaki Matsumoto, Hisayoshi Imanishi, Mika Yamanaka-Takaichi, Masateru Hirae, Daisuke Tsuruta, Kozo Nakai

**Affiliations:** ^1^ Department of Dermatology, Graduate School of Medicine, Osaka City University, Osaka, Japan; ^2^ Department of Dermatology, Graduate School of Medicine, Osaka Metropolitan University, Osaka, Japan; ^3^ Merchandising Department, Science Co., Ltd, Osaka, Japan; ^4^ Department of Dermatology, Kochi Medical School, Kochi University, Kochi, Japan

**Keywords:** atopic dermatitis, ultrafine bubbles, interleukin-33, type 2 inflammation, shower

## Abstract

**Introduction:**

Atopic dermatitis (AD) is a common and relapsing skin disease characterized by skin barrier dysfunction, inflammation, and chronic pruritus. Both cutaneous barrier dysfunction and immune dysregulation are critical etiologies of the pathology of AD. Although various anti-inflammatory pharmacological agents, including cytokine inhibitors and signaling pathway blockers, have been developed recently, keeping the skin clean is of utmost importance in maintaining physiological cutaneous barrier function and avoiding an AD flare. Ultrafine bubbles (UFBs) are less than 1 μm in diameter and usually used to clean medical equipment. A UFB shower is expected to keep skin clean with attention to the temperature and strength of the shower.

**Methods:**

We examined the effects of a UFB shower on two mouse models of AD: Dermatophagoides farinae body (Dfb)- induced AD in NC/Nga mice and interleukin (IL)-33 transgenic (tg) mice. Each model comprised three groups: UFB shower-treated, normal shower-treated, and untreated. We evaluated the mice using a dermatitis score, scratching counts, histology, and the expression of inflammatory cytokines and skin barrier-related proteins.

**Results:**

In the Dfb-induced AD mouse model, clinical features improved markedly in the UFB shower-treated mice compared to other groups. IL-4 and IL-13 levels decreased in the skin of normal and UFB shower-treated mice. In addition, in the skin of UFB shower-treated mice, the expression levels of skin barrier-related proteins were increased compared to normal showertreated mice. However, we found no significant differences in IL33tg mice.

**Discussion:**

These results suggest that UFB shower can recover the skin barrier function and improve skin inflammation, especially in conditions such as extrinsic AD.

## 1 Introduction

Atopic dermatitis (AD) is a common chronic inflammatory disease characterized by purpuric relapsing skin erythema and papules, but the phenotype is heterogeneous and partially depends on the patient’s age, race, and lifestyle. Therefore, the pathophysiology of AD is complex and multifactorial. Nevertheless, recent studies have established genetic factors, deranged skin microbiome, skin barrier dysfunction, and immune dysregulation as critical etiologies of AD. These factors are interconnected and influence each other to initiate and aggravate AD symptoms. Skin barrier dysfunction in AD can manifest as lower expression of terminal keratinization-related markers, cell junction-related markers, and cytoskeletal markers filaggrin, loricrin, claudin-1, and Kazrin. Skin barrier dysfunction may potentiate the penetration of harmful agents. Allergens or helminths have been reported to disrupt epithelial barriers and induce type 2 responses (1). Allergens exacerbating AD are animal dander, pollution, and house dust mites (HDMs) (2). Mite antigen has been suggested to play important roles in the onset and/or development of AD, and an AD model has been established in NC/Nga mice using Dermatophagoides farinae (3). Allergens derived from HDMs have enzymatic activity and destroy tight junctions, deteriorating the skin barrier function in patients with AD (4). Damaged keratinocytes produce epidermal alarmins, such as interleukin (IL)-33, IL-25, and Thymic stromal lymphopoietin (TSLP), which activate the dendritic cells (DCs) and type 2 innate lymphoid cells (ILC2s) that produce IL-5 and IL-13. These, in turn, activate eosinophils and Th2 cells (5). AD lesions are primarily Th2-driven with the overproduction of important Th2 cytokines and chemokines, including IL-4, IL-5, IL-13, and CC-chemokine (CCL) 22 (6, 7). The contributions of the Th1, Th17, and Th22 axes vary depending on the AD phenotype. In addition, local Th2 polarization with skin barrier impairment facilitates dysbiosis (8). Therefore, although various anti-inflammatory pharmacological agents, including cytokine inhibitors and signaling pathway blockers, have been developed recently, bathing and showering is the most important treatment to maintain physiological cutaneous barrier function and avoid an AD flare.

In general, bathing and showering are encouraged to remove infectious pathogens, antigens, allergens, and pollution from the skin of AD patients. Sweat is necessary to maintain skin homeostasis because it includes natural moisturizing factors and antimicrobial peptides, among other constituents. Some AD patients are reported to sweat less than healthy controls (9–11). However, sweat is also known to be an antigen and cause itching. AD patients exhibit positive reactions to sweat antigens in a histamine release test, and MGL_1304, a fungal protein derived from *Malassezia globosa*, is reported to be a major sweat antigen (12). Thus, showering after sweating has been suggested to relieve AD symptoms (13, 14). In addition, bathing and showering ameliorate skin barrier abnormalities, including decreased filaggrin, ceramides, and antimicrobial peptides, as well as disordered tight junctions in AD (15). An appropriate water temperature during bathing or showering could recover skin barrier function (16). The use of soap or detergent during bathing or showering may also improve AD symptoms (17). Another study showed beneficial effects of skin care using shower therapy for AD patients during elementary school (13). These reports indicate the importance of bathing or showering and necessitate the development of further novel methods or instruments for bathing or showering AD patients.

Ultrafine bubbles (UFBs) are tiny bubbles in water with diameters of several tens to hundreds of nanometers. UFBs were discovered in Japan during research on microbubbles. Many applications of UFBs in various engineering fields have been reported (18). UFBs have also been used in showering or bathing. In a recent study comparing the UFB shower with a tap-water shower, the UFB shower resulted in a significantly higher stratum corneum water content 15 and 30 min after the shower (19). However, the effectiveness of UFB showers for skin diseases has not been studied. In the present study, we examined the effects of UFB shower on mouse models of two types of AD: Dfb-induced AD and IL-33 transgenic (tg) mice.

## 2 Materials and methods

### 2.1 Mice

#### 2.1.1 Dfb-induced AD in NC/Nga mice

Nine-week-old female NC/Nga mice were purchased from Japan SLC, Inc. (Hamamatsu, Japan). The mice were maintained under conventional conditions in our laboratory or the breeding room of Oriental Bio Service Co., Ltd., Kobe BM Laboratory. One week after arriving, mice were anesthetized by injection of medetomidine chloride (Nihon Zenyaku Kogyo Co., Ltd., Koriyama, Japan), midazolam (Sandoz K.K., Tokyo, Japan), and butorphanol tartrate (Meiji Seika Pharma Co., Ltd., Tokyo, Japan). The hair on their back was shaved using an electric shaver and removed with skin-hair remover (Kanebo, Tokyo, Japan). Mice were treated topically with Dfb extract and hair removal performed twice weekly for 3 weeks (20). Procedures performed in this study were approved by the ethics committee for animal experiments at Osaka City University.

#### 2.1.2 IL-33 transgenic mice

The mouse line hK14mIL33tg (IL33tg) expressing IL-33 driven by a keratin-14 promoter was generated as described previously (21). Eight-week-old female C57BL/6J mice were purchased from Trans Genic, Inc. Mice were maintained under specific pathogen-free conditions in the breeding room of Oriental Bio Service Co., Ltd., Kobe BM Laboratory. Marked dermatitis was observed at 25 weeks of age, and shower treatment was started at 27 weeks of age. All animal studies were designed in accordance with the International Guiding Principles for Biomedical Research Involving Animals published by the Council for the International Organization of Medical Science.

### 2.2 Shower treatment and sampling

After we confirmed that dermatitis developed in the mice, we divided each model into three groups: an untreated group, normal shower group, and UFB shower group. We showered the back of each mouse in the treatment groups for 1 minute every day for 7 to 14 days. One IL33tg mouse died during the UFB shower treatment. In Dfb-induced AD mice, we did not use topical treatment of Dfb after shower treatment was started. In Dfb-induced AD mice, we sampled the back skin before and after shower treatment. In IL33tg mice, we sampled the facial skin and back skin after shower treatment. The skin was divided into three samples: one fixed in formalin, one that was cryopreserved, and one that was processed for RNA extraction.

### 2.3 Dermatitis scoring

#### 2.3.1 Dfb-induced AD model

The severity score (0 to 15) was defined as the sum of the individual scores graded as 0 (none), 1 (mild), 2 (moderate), or 3 (severe) for each of five symptoms: pruritus, erythema, edema/papulation, excoriation, and scaling/dryness (22).

#### 2.3.2 IL33tg mice

The severity score (0 to 12) was defined as the sum of the individual scores graded as 0 (none), 1 (mild), 2 (moderate), or 3 (severe) for each of four symptoms: loss of back hair, blepharitis, perinasal inflammation, tail inflammation.

### 2.4 Scratching behavior

Scratching behavior was defined as rapid, repetitive, back-and forth movement of the hindlimb. We identified scratching behavior on a recorded video file (23). One hour after placing the mice in the gauge for filming, we observed the mouse’s behavior for 30 minutes to 1 hour.

### 2.5 Histology and immunohistochemistry

The tissues embedded in paraffin were resected and 4-µm-thick sections stained with hematoxylin and eosin (H&E) for histopathological observation. The thickness of the epidermis was measured by NDP view 2 (Hamamatsu Photonics Co., Ltd.).

Immunohistochemistry was performed on paraffin-embedded skin using the following antibodies: rabbit anti-filaggrin (abcam, ab24584; 1:5000), rabbit anti-loricrin (abcam, ab24722; 1:5000), rabbit anti-involcrin (abcam, ab53112; 1:500), rabbit anti-claudin1 (Invitrogen, 51-9000; 1:200), and rabbit anti-Kazrin (cosmo, 115721-AP; 1:500). Sections were incubated overnight at 4°C with the primary antibodies, followed by incubation with horse anti-rabbit IgG from the ImmPRESS^®^Universal Polymer Kit (Vector Laboratories, MP-7401). Finally, we used the ImmPACT^®^ DAB EqV Substrate Kit (Vector Laboratories, SK-4103).

### 2.6 Real-time PCR

RNA was extracted using RNAiso plus (TaKaRa) and reverse transcription performed using PCR Thermal Cycler Dice Touch (TaKaRa). RT-PCR was carried out using Taqman Fast on a 7500 real-time PCR system (Applied Biosystems). Threshold cycles for each transcript were normalized to GAPDH (ΔCt). Calibrations and normalizations used the 2^−ΔΔCt^ method, where ΔΔCt = [(Ct (target gene) − Ct (reference gene)] − [Ct (calibrator) − Ct (reference gene)]. GAPDH was used as the reference gene.

The following primers were used: Mm99999915_g1 (Glyceraldehyde-3-phosphate dehydrogenase, GAPDH), Mm00445259_m1 (Interleukin 4, IL-4), Mm00439646_m1 (Interleukin 5, IL-5), Mm00434204_m1 (Interleukin 13, IL-13), Mm00439618_m1 (Interleukin 17, IL-17), Mm01716522_m1 (Filaggrin, Fig), Mm01962650_s1 (Loricrin, Lor), Mm00515219_s1 (Involcrin, Ivl), Mm01342184_m1 (Claudin-1, Cldn1), Mm01157588_m1 (Thymic Stromal Lymphopoetin, TSLP), Mm00511522_m1 (Serine Peptidase Inhibitor Kazal Type 5, Spink5), Mm00448862_s1 (Small Proline Rich Protein 3, Sprr3), Mm00470361_m1 (Transmembrane protein 79, Tmem79), and Mm00513780_m1 (Kazrin, Kaz).

### 2.7 Statistical analysis

Statistical analyses were performed using IBM SPSS Statistics. Continuous variables were summarized as mean ± standard error (SE). The data were compared by the Student t-test. P-values < 0.05 were considered significant. P-values < 0.05 were considered significant.

## 3 Results

### 3.1 UFB shower treatment improved the dermatitis score in Dfb-induced AD mice

**Figure 1:**
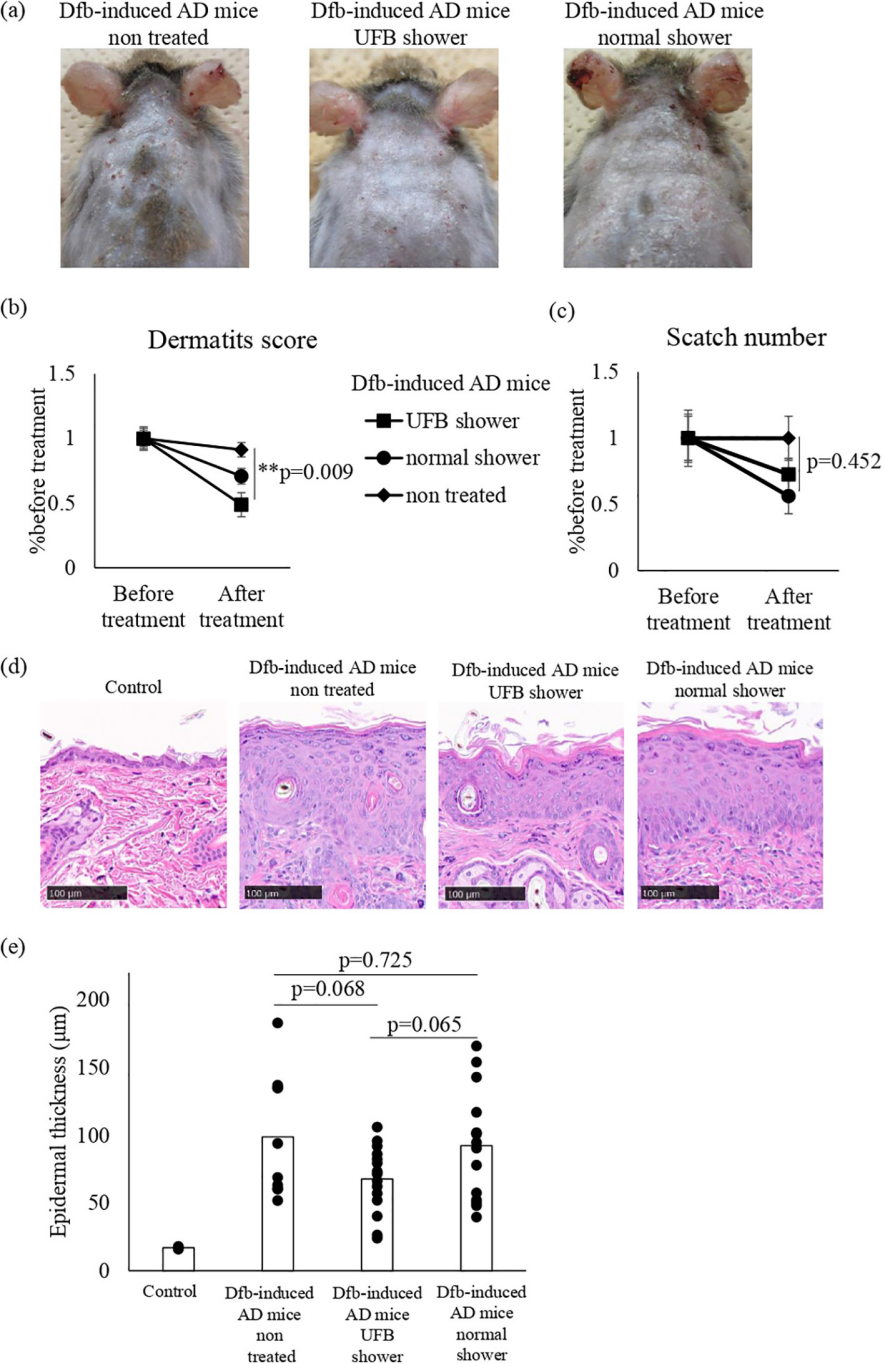
Changes in the dermatitis score, scratch number, and skin thickness in Dfb-induced AD mice after shower treatment. **(A)** The mice were treated with a UFB shower or normal shower for 7-14 days. **(B)** The dermatitis score was defined by five symptoms (pruritus, erythema, edema/papulation, excoriation, and scaling/dryness). **(C)** The scratch number was counted for 30-60 minutes and compared to the number before treatment. **(D)** The back skin of the mouse was collected and sectioned to measure skin thickness by NDP view 2. Scale bars: 100 μm. **(E)** The average of three areas of thickened epidermis. Dfb, Dermatophagoides farinae body; AD, atopic dermatitis; UFB, ultrafine bubble; **p < 0.01. Data represent means ± SE.

Before shower treatment, we ensured the presence of erythema, papules, thick scales, and scratch marks on the back skin of Dfb-induced AD mice. Compared to untreated mice, erythema and papules were reduced in both UFB shower-treated mice and normal shower-treated mice, and the scale and erythema in UFB shower-treated mice were markedly improved compared to normal shower-treated mice (**Figure 1A**). The dermatitis score was significantly reduced in UFB shower-treated mice compared to untreated mice (p=0.009, **Figure 1B**). There was no significant difference in the scratch number among the UFB shower-treated group, the normal shower-treated group, and the non-treated group (p=0.452, **Figure 1C**). Histologically, the thickness of the epidermis and granular layer appeared to be decreased in the UFB shower-treated group, but no significance was found (**Figures 1D, E**). These results suggest that the UFB shower mildly ameliorated clinical symptoms without an apparent reduction in pruritis or clear histological change in the lesion skin of Dfb-induced AD mice.

### 3.2 UFB shower treatment downregulated the expression of AD-related inflammatory cytokine genes and upregulated the expression of skin barrier-related factor genes in the skin of Dfb-induced AD mice in RNA sequence

**Figure 2:**
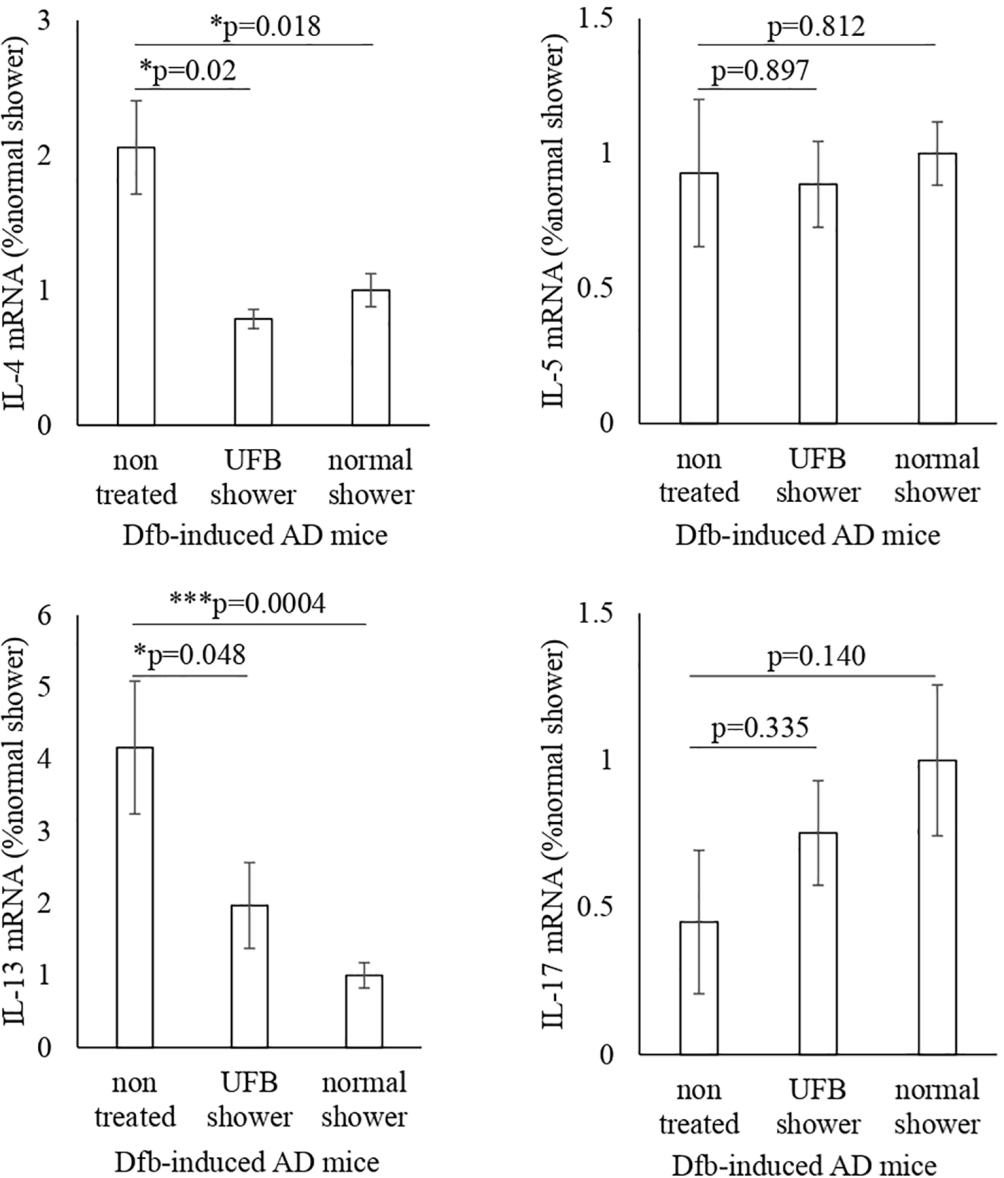
Changes in inflammatory cytokines and RNA-seq in Dfb-induced AD mice after shower treatment. The IL-4, IL-5, IL-13, and IL-17 gene expression was measured by RT-PCR. Data represent means ± SE. N = 9-15. Dfb, Dermatophagoides farinae body; AD, atopic dermatitis; IL, interleukin; UFB, ultrafine bubble; *p < 0.05, ***p < 0.001.

**Figure 3:**
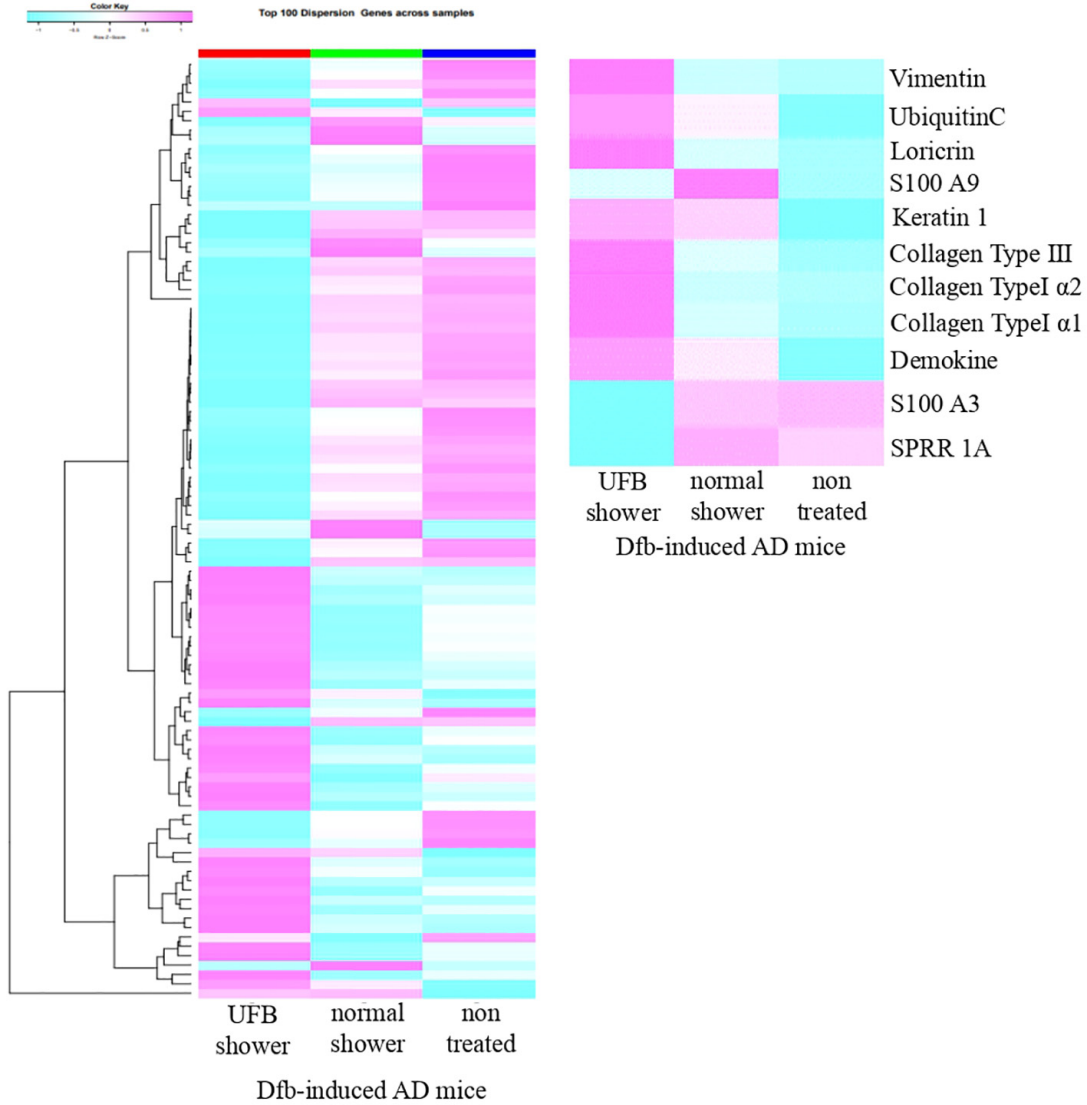
The RNA-seq result for one sample from each of three groups. The proteins that play a role in the skin's barrier function increased in mice treated with the UFB shower.

We examined inflammatory cytokines in the skin of Dfb-induced AD mice using semi-quantitative RT-PCR (**Figure 2**). Compared to untreated mice, decreased mRNA levels of IL-4 and IL-13 were observed in the skin of both the UFB shower-treated group and normal shower-treated group. The mRNA levels of IL-5 and IL-17 were not decreased by UFB shower treatment or normal shower treatment. To investigate the effect of UFB shower treatment on the AD model, we performed RNA sequence analysis (**Figure 3**). This data has been registered to DNA Data Bank of JAPAN (DDBJ; No. DRR621560, DRR621561, DRR621562). As shown in **Figure 3**, RNA expression of vimentin, ubiquitin C, loricrin, keratin 1, and some collagens was increased in the skin of Dfb-induced AD mice by UFB shower treatment. These results imply that UFB shower treatment could restore the skin barrier function in the skin of Dfb-induced AD mice.

### 3.3 UFB shower treatment upregulated the expression of skin barrier-related factors in the skin of Dfb-induced AD mice in semi-quantitative RT-PCR

**Figure 4:**
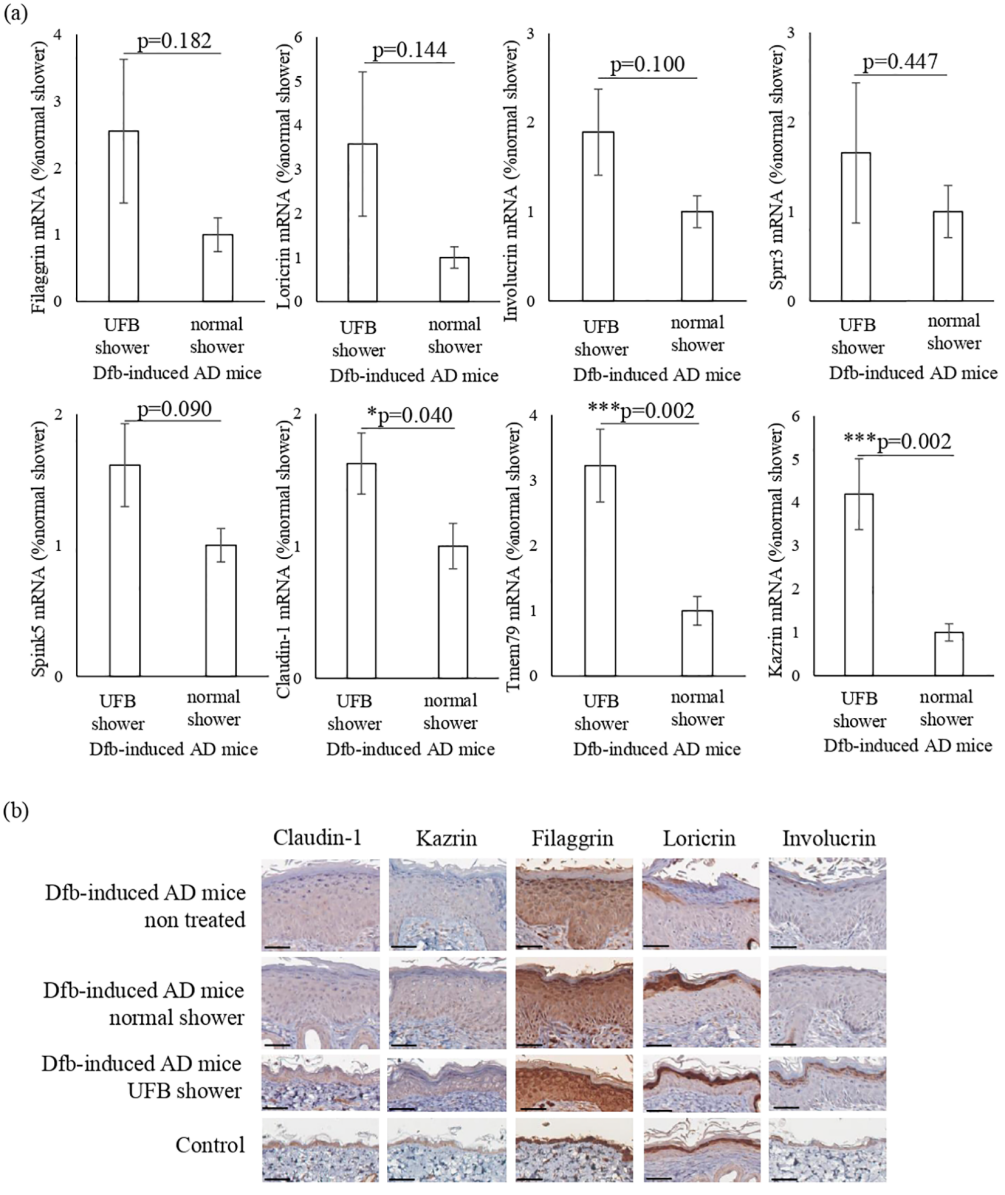
Changes in proteins that play a role in the barrier function of the skin. **(A)** The expression of proteins distributed from the stratum corneum to the stratum spinosum was measured at the mRNA level. Claudin-1, which forms tight junctions, Tmem79, which is mainly in the granular layer, and Kazrin, which is mainly in the spinous layer, were significantly increased by UFB shower treatment. Data represent means ± SE. N = 9-15. *p < 0.05, ***p < 0.001. **(B)** Expression of filaggrin, loricrin, involucrin, claudin-1, and Kazrin in the back skin was examined by immunohistochemical staining. Scale bars: 50 μm.

Based on the RNA sequence results, we hypothesized that the UFB shower affects skin barrier molecules. We performed semi-quantitative RT-PCR analysis of genes related to skin barrier function: filaggrin, loricrin, involucrin, claudin-1, transmembrane protein 79 (Tmem79), serine peptidase inhibitor Kazal type 5 (Spink5), small proline rich protein 3 (Sprr3), and Kazrin (**Figure 4A**).

In the skin of UFB shower-treated Dfb-induced AD mice, the expression levels of claudin-1 (p=0.04), Tmem79 (p=0.002), and Kazrin (p=0.002) were significantly increased (**Figure 4A**). The expression levels of filaggrin, loricrin, involucrin, Sprr3, and Spink5 in the skin were not different between the UFB shower-treated group and the normal shower-treated group (**Figure 4A**). Immunohistochemical analysis revealed that UFB shower treatment increased the expression of filaggrin and involucrin protein, and that both UFB shower treatment and normal shower treatment increased loricrin protein expression in the skin of Dfb-induced AD mice (**Figure 4B**). The protein expression of claudin-1 and Kazrin was not altered in the skin of Dfb-induced AD mice after shower treatment. These results suggest that UFB shower treatment increased the mRNA expression of claudin-1 and Kazrin but did not increase the expression of these proteins in the skin of Dfb-induced AD mice. UFB shower treatment increased the post-translational expression of filaggrin, loricrin, and involucrin in the skin of Dfb-induced AD mice.

### 3.4 UFB shower treatment did not alter AD-related inflammatory cytokines and skin barrier-related factors in IL33tg mice

**Figure 5:**
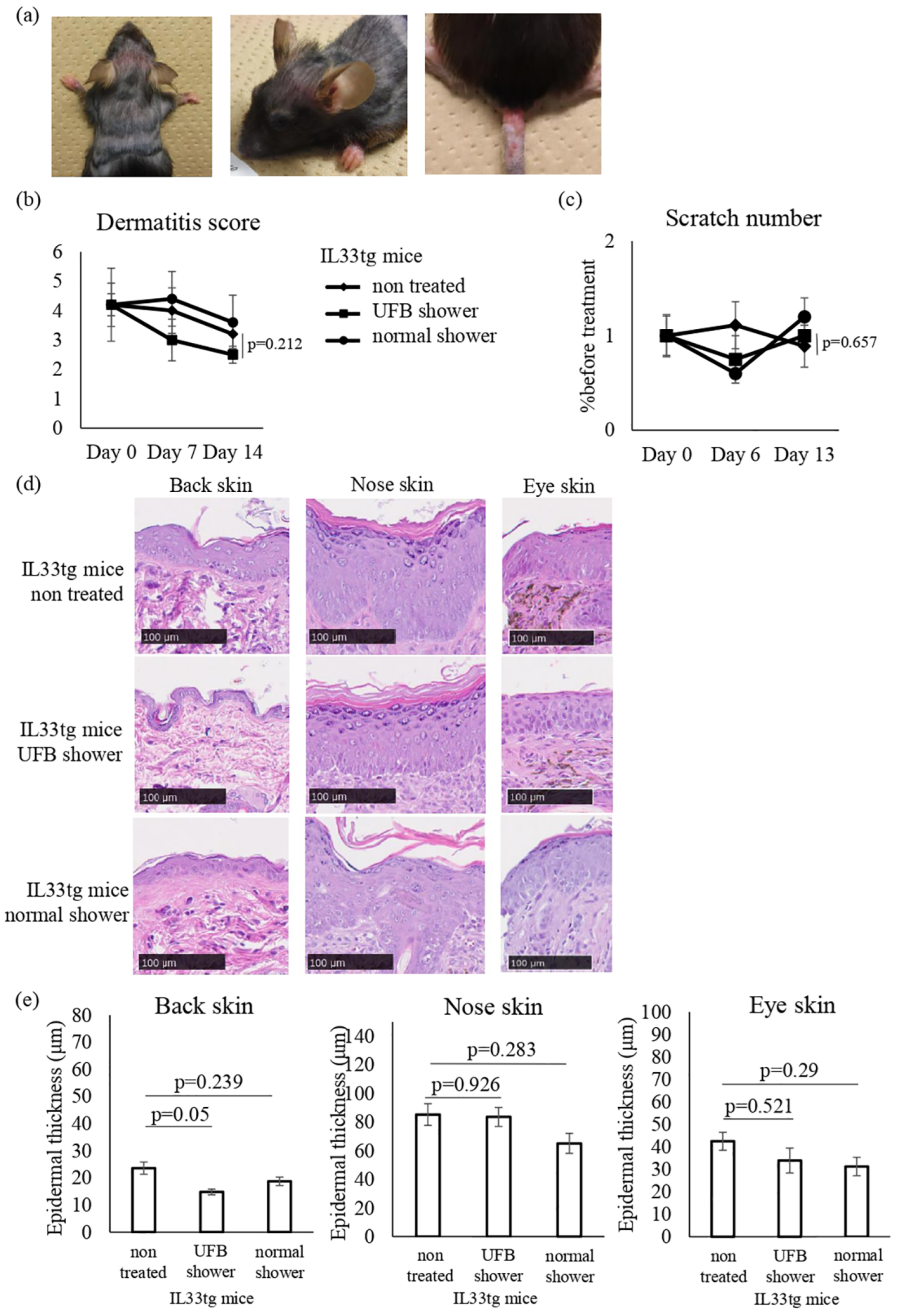
Changes in the dermatitis score, scratch number, and skin thickness in IL-33 transgenic mice. **(A)** Mouse before shower treatment. Note the erythema, edema, and erosion around the eyes, nose, and at the base of the tail, as well as hair loss on the back. **(B)** The dermatitis score was defined by five symptoms (pruritus, erythema, edema/papulation, excoriation, and scaling/dryness). **(C)** The scratch number was counted for 30-60 minutes and compared to the number before treatment. **(D)** The skin of the mouse was collected and sectioned to measure skin thickness by NDP view 2. Scale bars: 100 μm. **(E)** The average of three areas of thickened epidermis. Data represent means ± SE.

The IL33tg mouse is a genetic IL-33-initiated model of AD (24). We examined the effects of UFB shower treatment on IL33tg mice. An AD-like condition was present in the periocular, perirhinal, and tail skin of IL33tg mice as reported previously (**Figure 5A**). Some IL33tg mice had hair loss, but there was no obvious erythema on the back of the skin. There was no significant difference in the dermatitis score and the scratch number among the UFB shower-treated group, the normal shower-treated group, and the non-treated group (**Figures 5B, C**). Furthermore, normal and UFB shower-treated groups did not significantly differ from non-treated group in the thickness of the skin on the back, eyes, and nose in IL33tg mice (**Figures 5D, E**).

**Figure 6:**
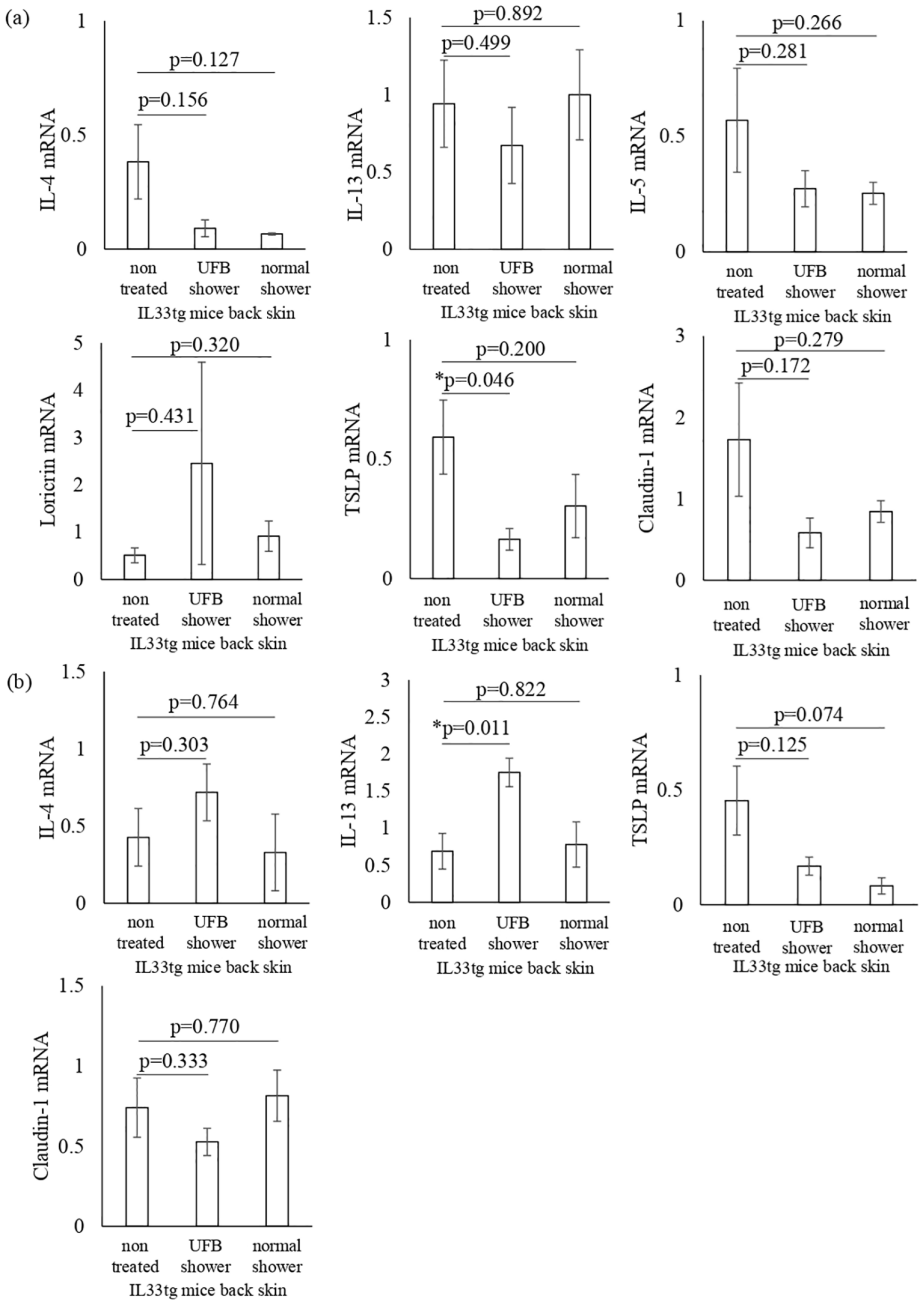
Changes in inflammatory cytokines and proteins that play a role in the skin's barrier function in IL-33 transgenic mice. Cytokines and proteins distributed from the stratum corneum to the stratum spinosum in the back skin **(A)** and facial skin **(B)** were measured at the mRNA level. There was no significant difference between the three groups. Data represent means ± SE. N=4-5. *p < 0.05..

Next, we examined the mRNA expression of AD-related inflammatory cytokines and skin barrier-related factors in both the back and facial skin of IL33tg mice. The levels of TSLP mRNA were significantly decreased in the back skin of IL33tg mice after UFB shower treatment (p=0.046, **Figure 6A**). UFB shower treatment did not alter the expression levels of barrier function proteins, such as claudin-1 and loricrin, in the back skin of IL33tg mice (**Figure 6A**). In the facial skin of IL33tg mice, the expression level of IL-13 was increased by UFB shower treatment (P=0.011, **Figure 6B**). Overall, no marked beneficial effects of UFB shower treatment were observed in IL33tg mice.

## 4 Discussion

In the present study, we elucidated the effects of UFB shower treatment on AD mouse models. We examined two types of AD mice, Dfb-induced AD mice in which the HDM antigen disrupts the epidermal barrier with a subsequent immune response plus inflammation, and IL33tg mice in which IL-33 initiates an immune response with the subsequent inflammatory cascade. In Dfb-induced AD mice, UFB shower treatment improved the dermatitis score, downregulated the gene expression of AD-related inflammatory cytokines (IL-4 and IL-13), and upregulated skin barrier-related molecules. However, UFB shower treatment did not result in significant changes in IL33tg mice.

**Figure 7:**
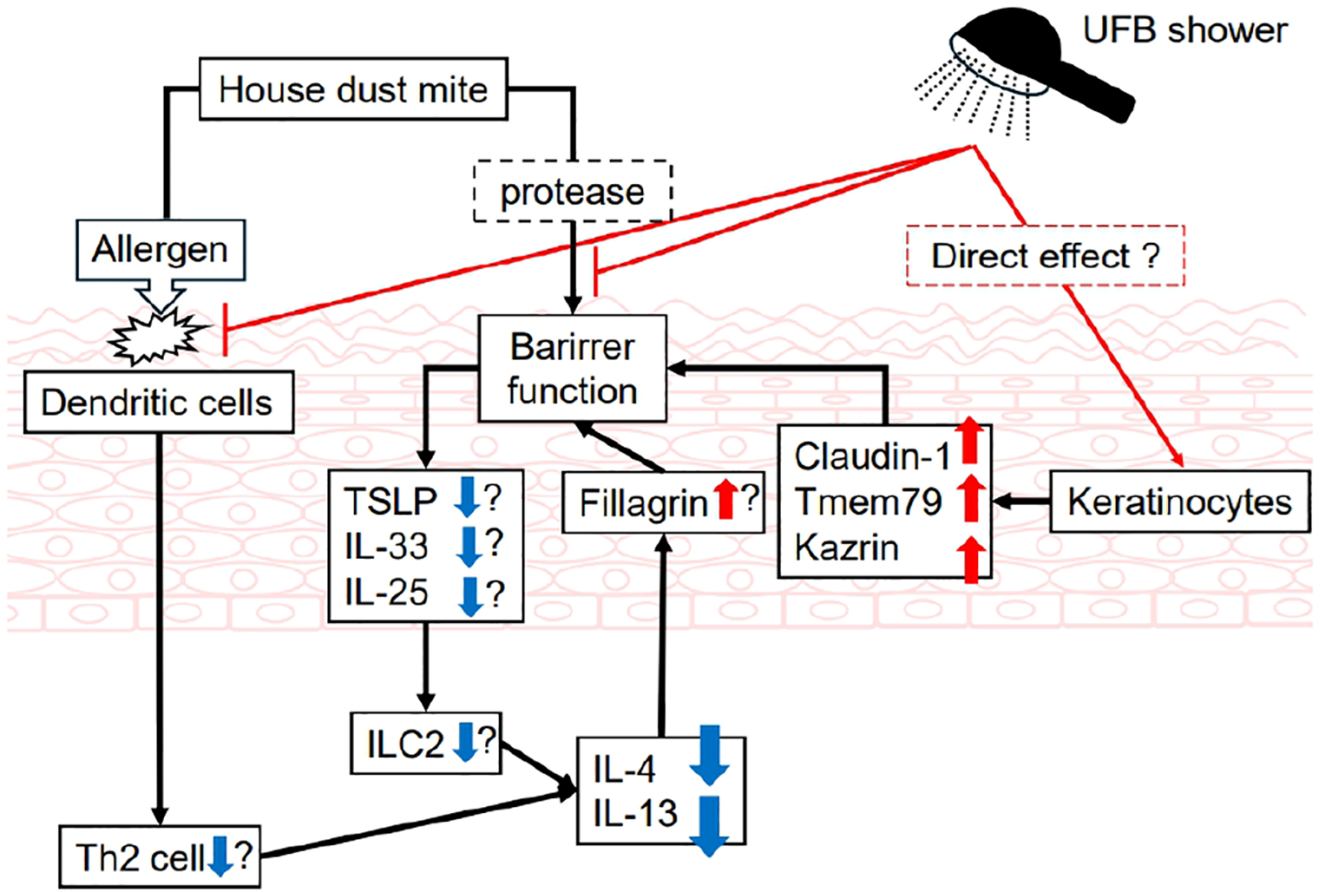
Schematic diagram of UFB shower’s effects in atopic dermatitis skin. Blue arrows depict decreasing levels, and red arrows indicate increasing levels. Question mark indicates uncertain but expected effects. UFB, Ultra fine bubble; IL, interleukin; ILC, Innate lymphoid cells; TSLP, Thymic Stromal Lymphopoetin; Tmem79, Transmembrane protein 79.

The causes of AD are complex and multifactorial, and the pathophysiology of AD involves a complex interplay between a dysfunctional epidermal barrier, skin microbiome abnormalities, and immune dysregulation predominantly skewed towards type 2 with a background of genetic risk factors (25). With these conditions, allergens can synergistically aggravate AD, and removing these harmful factors by bathing or showering may be fundamental to the treatment of AD. As UFB has high cleaning efficiency across various industrial fields, UFB shower treatment was suggested to potentially improve AD by completely removing allergens. Especially, there are many allergens of house dust mite and house dust mite produces proteases that impair the skin barrier function (26). Epicutaneous application of house dust mite allergens induces expression of TSLP in nonlesional skin of atopic dermatitis patients (27). It is possible that both UFB and normal shower can remove house dust mite and improve skin barrier function. However, dermatitis score is significantly improved only in UFB shower treatment group. In addition, we found novel biological effects of UFB shower on the skin of AD mouse models. UFB shower treatment increased the mRNA expression of claudin-1, Tmem79, and Kazrin and increased the post-translational expression of filaggrin, loricrin, and involucrin in the skin of Dfb-induced AD mice. Moreover, the expression levels of inflammatory cytokines IL-4 and IL-13, as well as dermatitis scores, significantly improved after UFB shower treatment in Dfb-induced AD mice. Thus, UFB shower treatment could be a novel beneficial treatment for AD by improving skin barrier function. Similarly, the effects of UFB shower treatment on skin barrier function was supported by a previous report of an increased adsorption rate of ceramides and barrier proteins in the skin and increased skin hydration (19). Claudin-1 and Tmem79 are present in tight junctions, and Kazrin is present in the stratum spinosum. The fine bubbles of the UFB could improve skin barrier function in AD by removing allergens and stimulating directly or indirectly the deeper layers of the epidermis, promoting the synthesis of these proteins (**Figure 7**). As we treated the Dfb-induced AD mice with UFB shower for only a few weeks, its effects were not obvious; some inflammatory cytokines were not suppressed, histological epidermal thickness was not changed, and scratch number was not reduced. However, we presume that UFB shower treatment for a few months could improve these observations.

We could not find beneficial effects of UFB shower treatment on IL33tg mice except for a reduction in TSLP mRNA expression in the back skin. Unlike Dfb-induced AD mice, the AD inflammation in the IL33tg mice is not initiated by allergens, but by genetically enhanced IL-33 expression. IL-33 is known to downregulate the expression of filaggrin and to disrupt the skin barrier (28).

In IL33tg mice, the skin barrier disruption by IL-33 may be a concomitant phenomenon, and AD inflammation mainly depends on innate immune responses that are mediated by type 2 innate lymphoid cells with basophils (24). Our results suggest that UFB shower treatment is not effective for IL-33-induced skin barrier disruption and IL-33-initiated AD inflammation. Alternatively, inflammatory signals were spontaneously activated without allergens, and a few weeks of UFB shower treatment may be too short and weak an intervention to improve AD inflammation in IL33tg mice.

Another controversial result in the UFB shower-treated IL33tg mice may be the differences in some mRNA expression levels between the back skin and facial skin, particularly for IL-4 and IL-13. UFB shower treatment appeared to decrease IL-4 expression in the back skin but appeared to increase expression in the face skin of IL33tg mice. IL-13 expression was significantly increased at the mRNA level in the face skin of IL33tg mice after UFB shower treatment. Thus, the effects of UFB shower treatment may depend on the area of the skin lesion in IL33tg mice.

As AD is associated with complex pathophysiology and heterogeneous skin manifestations, it is difficult to determine the effects of UFB shower treatment in all types of AD using only these two models. Therefore, further studies are needed in which showering time, duration, shower head-skin surface distance, and skin area are measured.

In summary, we examined the effects of UFB shower treatment on two types of AD mice. UFB shower treatment improved clinical symptoms, possibly by recovering skin barrier function and downregulating the subsequent gene expression of AD-related inflammatory cytokines in an allergen-induced AD model. However, UFB shower treatment did not result in beneficial effects on AD inflammation in genetically initiated AD mice. Thus, UFB shower treatment could be a novel treatment in some types of AD.

## Data Availability

The datasets presented in this study can be found in online repositories. The names of the repository/repositories and accession number(s) can be found below: DRR621560, DRR621561, DRR621562 (DNA Data Bank of JAPAN; DDBJ- https://ddbj.nig.ac.jp/search/).
